# The Risk of Developing Lymphoma among Autoimmune Thyroid Disorder Patients: A Cross-Section Study

**DOI:** 10.1155/2022/4354595

**Published:** 2022-06-03

**Authors:** Mubarak Al-Mansour, Alaa Fawzi Maglan, Meral Khalid Altayeb, Laila Ali Faraj, Esraa Aman Felimban, Syed Sameer Aga, Mohammad Anwar Khan

**Affiliations:** ^1^Princess Noorah Oncology Center, National Guard Health Affairs (NGHA), King Saud Bin Abdul Aziz University for Health Sciences (KSAU-HS), King Abdullah International Medical Research Centre (KAIMRC), King Abdulaziz Medical City, Jeddah, Saudi Arabia; ^2^Department Medical Education, College of Medicine, King Saud Bin Abdul Aziz University for Health Sciences (KSAU-HS), King Abdullah International Medical Research Centre (KAIMRC), National Guard Health Affairs (NGHA), King Abdulaziz Medical City, Jeddah, Saudi Arabia; ^3^Department of Basic Medical Sciences & Quality Unit, College of Medicine, King Saud Bin Abdul Aziz University for Health Sciences (KSAU-HS), King Abdullah International Medical Research Centre (KAIMRC), National Guard Health Affairs (NGHA), King Abdulaziz Medical City, Jeddah, Saudi Arabia

## Abstract

**Background:**

Graves' disease (GD) and Hashimoto's thyroiditis (HT) are the most common types of autoimmune thyroid diseases (AITD), and both are characterized by the infiltration of lymphocytes into the thyroid gland. Moreover, autoimmune diseases like HT have a higher risk of developing lymphoma. This study is aimed at assessing the prevalence and association of lymphoma in patients with AITD.

**Methods:**

This cross-sectional study was conducted in King Abdulaziz Medical City, Jeddah, Saudi Arabia. Data were gathered from the medical records of patients aged 18 years or older who developed AITD. A total number of 140 medical records were collected, and 72 patients were included after applying in exclusion criteria. Data on the subtype, clinical-stage, treatment modality, patient status, remission, and relapse were collected for patients who developed lymphoma.

**Results:**

Among 72 patients who developed AITD, HT was diagnosed in 58 (80.6%) patients and GD in 14 (19.4%). Five (7%) patients were diagnosed with lymphoma all of whom had a history of HT. The subtypes of lymphoma were diffuse large B-cell lymphoma (DLBCL 3; 4.2%), follicular lymphoma 1 (1.4%), and Hodgkin's lymphoma 1 (1.4%).

**Conclusion:**

The prevalence of PTL in patients with AITD, specifically HT, was 7%. Most patients developed NHL, with DLBCL being the most common subtype. The onset of lymphoma in this study was lower than reported in the literature. All patients with PTL had HT in their backgrounds. Further national studies are warranted to explore the relationship between the two diseases to provide more insight into the comprehension of this association.

## 1. Introduction

The thyroid gland is an essential endocrine organ that produces triiodothyronine (T3) and thyroxine (T4) or “thyroid hormones” [1]. These hormones regulate basal metabolic rate, cardiac output, normal growth, and nerve development [2]. Thus, any disorder affecting the thyroid gland can have major effects on the human system, such as autoimmune thyroid disease (AITD) [3]. Graves' disease (GD) and Hashimoto's thyroiditis (HT) are the two most frequent types of AITD, and both diseases are characterized by lymphocyte infiltration in the thyroid gland [3].

In HT, an autoreactive T helper cell initiates the disease by activating cytotoxic T and B cells [4]. Thyroid peroxidase and thyroglobulin antibodies are produced by this activation, resulting in the destruction of follicular cells and hypothyroidism [5]. In GD, CD4+ cells activate B lymphocytes, causing them to produce self-antibodies known as thyroid-stimulating immunoglobulin (TSI) [6], which mimics the effect of thyroid-stimulating hormone, increasing the production of thyroid hormones, leading to hyperthyroidism [7].

The prevalence of thyroid lesions, including AITD, is affected by multiple factors, including age, gender, genetics, smoking, iodine intake, radiation exposure, and environmental toxins [8, 9]. AITDs affect up to 5% of the general population and are mostly seen in women between 30 and 50 years of age. The annual incidence of HT worldwide is estimated to be 0.3–1.5 cases per 1000 persons, whereas GD is estimated at 5 per 10,000 people [10]. Meanwhile, in a study conducted in the western region of Saudi Arabia, the prevalence was 7.6% for HT and 0.9% for GD in a total of 163 specimens [11].

Many studies have demonstrated that a dysfunctional immune system that attacks its cells can lead to cancer [12–14]. Patients with autoimmune diseases such as HT or GD have a higher risk of developing lymphoma [12–14]. Lymphoma is a malignant neoplasm of the lymphatic system that is categorized according to the origin of the cells and stage of differentiation. The two main types of lymphoma are Hodgkin's lymphoma (HL), which is derived from B cells, and non-Hodgkin's lymphoma (NHL) which is derived from B cells and T cells [10]. NHL is the most common type in adults [15]. According to the Saudi Cancer Registry of 2014, NHL and HL are among the top ten cancers in incidence among Saudis [16]. Xie et al. showed that primary thyroid lymphoma (PTL) comprises approximately 5% of all malignant thyroid tumors and fewer than 3% of all extranodal lymphomas, intrinsically related to HT [17].

Most studies on the association between AITD and PLT involve case series and case reports; however, large-scale retrospective or cross-sectional studies are limited. To the best of our knowledge, no study from Saudi Arabia has explored or assessed the prevalence and association between the two diseases.

Therefore, this study is aimed at determining the prevalence of PTL among patients with AITD, stressing the significance of physicians keeping lymphoma in mind in patients with a clinical history of AITD.

## 2. Materials and Methods

This cross-sectional study was conducted from January 2010 to September 2020 at the Princess Norah Oncology Center (PNOC) in King Abdulaziz Medical City (KAMC), Jeddah, Saudi Arabia. Appropriate ethical approval as per the Helsinki protocol was obtained prior to conducting this study via the Institutional Research Board (IRB). This study was approved by the Institutional Review Board of the King Abdullah International Medical Research Centre (KAIMRC), a research wing of KSAU-HS, Jeddah. The study protocol was approved by the Institutional Review Board (IRB) of KAMC bearing the ethical approval number of IRBC/0797/19.

The study included all adult patients (≥18 years) with AITD and biopsy-proven lymphoma. We excluded patients who were diagnosed with lymphoma at KAMC but had their treatment elsewhere.

The data collection sheet included demographic data, the type of AITD, and whether the patient had received any pharmacological or surgical treatment for AITD. We added the subtype, clinical stage, and treatment modality for patients who developed lymphoma, including chemotherapy, radiation therapy, and surgery.

### 2.1. Statistical Analysis

Data were entered into a Microsoft Excel worksheet and analyzed using the Statistical Package for Social Science (SPSS; version 22). Data entry was reviewed to ensure the accuracy of the data analysis. Qualitative variables are presented as frequencies and percentages, whereas quantitative variables are presented as mean ± standard deviation. We used unpaired Student's *t*-tests to compare the mean age with diverse independent variables. The clinical characteristics of the AITD types and treatments were compared using the Fisher's exact test for categorical variables. Statistical significance was set at *p* < 0.05.

## 3. Results

### 3.1. Patients' Characteristics

The patient characteristics are summarized in Table 1. At the Princes Norah Oncology Center, the total number of patients who had AITD from January 2010 to December 2020 was 140; 68 patients were excluded for the following reasons: their age of diagnosis was less than 18 years, undocumented details about the diagnosis, and patients diagnosed before January 2010. About 63 (87.5%) of the patients were female. The mean age of diagnosis was 43.24 years (SD 14.75). Most of the cases were aged between 30 and 49 years. Only 12 (16.7%) of the patients had a family history of AITD. HT was diagnosed in 58 (80.6%) patients and GD in 14 (19.4%) patients.

### 3.2. Treatment of AITD

Most patients (42; 58.3%) had either a hemi or total thyroidectomy, while the other patients (16; 22.2%) have only received medications such as levothyroxine propylthiouracil and methimazole. Four (5.6%) of the patients received both drugs and surgery. Five (6.9%) patients received drug and radioiodine therapy, and the rest of the patients (5; 6.5%) did not receive any treatment.

In GD, seven (10.9%) patients received methimazole, 2 (3.1%) received propylthiouracil, 2 (3.1%) received radioactive iodine (RAI) with levothyroxine, 1 (1.6%) rituximab, and 1 (1.6%) patient was treated with total thyroidectomy. In terms of HT, the majority of patients had thyroidectomy (41; 64.1%) with 32 (47%) patients needed levothyroxine afterward, 5 (7.8%) treated with levothyroxine alone as a hormone replacement therapy, and 5 (7.8%) with no treatment (Table 1).

### 3.3. Prevalence of PTL

The prevalence of PTL in patients with AITD was 7%, with NHL predominance (4 out of 5). Patients who developed PTL had a history of HT. There was a female predominance in both AITD patients and lymphoma patients.

### 3.4. Lymphoma

Lymphoma subtypes were diffuse large B-cell lymphoma (DLBCL) 2 (2.8%), follicular lymphoma 2 (2.8%), and HL 1 (1.4%) (Table 2).

#### 3.4.1. Case 1

A 49-year-old female was presented with suspicious primary thyroid cancer after a positron emission tomography (PET) scan. In November 2014, she underwent fine-needle aspiration (FNA), which revealed cytological atypia in the context of chronic lymphocytic thyroiditis. In August 2015, the patient had a total thyroidectomy with the left cervical lymph node excision. Flow cytometry of the lymph node biopsy showed classical HL stage IA, with no lymphovascular invasion and no extrathyroid extension. The thyroid tissue section showed a prominent nodular pattern with lymphoid aggregates and prominent Hurthle cells with no evidence of malignancy. The patient received chemotherapy, two cycles of ABVD (Adriamycin® (doxorubicin), Bleomycin, Vinblastine, Dacarbazine), and achieved remission after radiotherapy.

#### 3.4.2. Case 2

A 48-year-old female was presented with a history of HT. After FNA, which was highly suspicious of B-cell lymphoma, the patient underwent total thyroidectomy in September 2012. The biopsy showed autoimmune lymphocytic thyroiditis and Hurthle cell changes, and the final diagnosis was follicle center B cell lymphoma, grade 3. In September 2012, the patient received three cycles of R-CHOP (rituximab, cyclophosphamide, doxorubicin, vincristine, and prednisone) and then underwent involving-field radiation therapy (IFRT) in December 2012. She achieved complete remission with no relapses.

#### 3.4.3. Case 3

A 55-year-old female patient was presented with an enlarged thyroid gland and multiple lymph node enlargements. The FNA, which was done in June 2012, showed chronic lymphocytic thyroiditis in the right thyroid and reactive lymphoid tissue in the right cervical lymph node. She was diagnosed with HT and received levothyroxine. In July 2012, this patient underwent a cervical lymph node biopsy for suspicions of lymphoma. The immunophenotyping of the lymph node biopsy showed a mixture of T cells and B cell populations, and the final diagnosis was follicular lymphoma, stage IIA, grade 2. In August 2012, a bone marrow biopsy was done for staging workup and showed a normocellular active bone marrow with no evidence of lymphoma infiltration. The patient did not undergo any treatment for lymphoma, just watchful waiting.

#### 3.4.4. Case 4

A 39-year-old male patient with neck swelling who had a history of ileocecal cancer received a chemotherapy regimen for ileocecal cancer and underwent a hemicolectomy in January 2009. In March 2015, FNA of the left thyroid was done and revealed chronic lymphocytic thyroiditis. Hence, the patient was diagnosed with HT and received levothyroxine. One month later, cervical lymph node enlargement was noticed. Subsequently, a thyroid biopsy was taken and revealed DLBC in both the right and left thyroid. The treatment of lymphoma, in this case, was six cycles of R-CHOP. However, the patient received two cycles, and then, he refused to continue and went for radiotherapy in December 2015. In July 2018, the bone marrow biopsy showed normocellular marrow with trilineage hematopoiesis with no evidence of bone marrow involvement by lymphoma. The patient achieved remission with no evidence of residual masses in the thyroid or lymphadenopathy.

#### 3.4.5. Case 5

A 59-year-old female patient had a lump in her neck with nausea and decreased appetite. She sought medical advice and was informed about her hypothyroidism with thyroid enlargement along with a suspicious lesion. An ultrasound was done and showed multiple nodules in both lobes. A right hemithyroidectomy was done and revealed diffuse NHL on the top of HT. Her labs showed hypothyroidism along with positive antithyroid peroxidase. The PET\CT was done in September 2020 and showed significant uptake. There is right-sided neck lymphadenopathy extending down the superior right mediastinum. The treatment of lymphoma for this case is six cycles of R-CHOP.

### 3.5. Risk Factors

There was no significant association between the gender of the AITD patients and the AITD type (*p* = 0.238), AITD treatment (*p* = 0.102), lymphoma type (*p* = 0.498), lymphoma treatment (*p* = 0.430), and the presence of a family history of thyroid disease (*p* = 0.534) (Table 3). Age at diagnosis of AITD was significantly associated with AITD type (*p* = 0.021). On the other hand, there was no significant association between age at diagnosis of AITD and developing lymphoma (*p* = 0.291) (Table 4).

## 4. Discussion

PTL is an uncommon type of thyroid cancer that accounts for 1% to 5% of all thyroid cancers and 1% to 7% of all extranodal lymphomas [13, 18–20]. PTL is more common in women than men, with a peak in the seventh decade [18, 20, 21]. A palpable mass in the neck is the most common clinical manifestation of PTL, which can induce hoarseness, dyspnea, or dysphagia. Furthermore, weight loss, night sweats, and fever may be present [18]. DLBCL, which accounts for 50% to 70% of cases, and mucosa-associated lymphoid tissue (MALT) lymphoma, which accounts for 10% to 50% of cases, are the most prevalent histotypes [22]. Less typically characterized lymphomas include Hodgkin lymphoma, T-cell lymphoma, mantle cell lymphoma, small lymphocytic lymphoma, follicular lymphoma, and Burkitt lymphoma [18].

In our study, 58 (80.6%) of the patients had HT, and 14 (19.4%) had GD, indicating that HT is more prevalent than GD in patients with AITD. In comparison to a study conducted in Al-Madinah in 2014 on 292 thyroidectomy specimens, 12 (4.1%) were HT positive while only 1 (0.3%) was GD positive [23]. Another study conducted in the western region of Saudi Arabia found that out of 173 patients, 18 (11.31%) patients developed HT [24]. Most of the cases presented in this research were found to have a multinodular goiter. All complained of neck swelling with compressive symptoms such as dyspnea, voice changes, and dysphagia, which corresponds with a study conducted in the western coastal area where midline neck mass followed by compressive symptoms is the most common presentation [25].

The prevalence of PTL in our patients with AITD was 7%, with NHL predominance (4/5), which is slightly higher than the previously reported incidence; however, it is still within the global range [13, 18–20]. Patients who developed PTL had a history of HT (100%), which is the most significant risk factor for PTL, increasing the risk of PTL by 40 to 80 times [13]. A recent meta-analysis of 38 studies showed that 78.9% of the PTL cases had HT, 65.3% confirmed with antithyroid antibodies, 41.7% with clinical history, and 64% with pathology. On the other hand, some studies reported no evidence of HT in a series of patients with PTL [26–28]. These variations might be explained by the varied techniques used to detect HT. Individuals with HT may not exhibit all of the disease's signs and symptoms, so the prevalence may vary depending on the diagnostic criteria used [29].

Regarding the histological type, we found that the most common type was DLBCL, followed by follicular lymphoma and HL. The aforementioned meta-analysis demonstrated that the HT prevalence was significantly higher in MALT lymphoma than in DLBCL (*p* = 0.007) and in mixed DLBCL/MALT than in pure DLBCL (*p* = 0.002) [30]. Only one patient in our study developed HL, which is considered a rare subtype of PTL [31]. The correlation between marginal zone lymphomas and AI has been extensively observed. The activation of the nuclear factor–*κ*B pathway appears to be critical in this process, as persistent stimulation of B cells produced by autoantigens is likely to raise the probability of cumulative genetic events [32]. The majority of cases of PTLs are MALT type, which develops from HT and can advance to DLBCL, indicating that PTL might be considered a single entity with a homogenous etiopathogenesis based on this theory [33]. Despite the obvious association between HT and PTL, it is unknown whether the presence of lymphocytes in the thyroid provides tissue enabling lymphoma to grow or whether persistent activation of lymphocytes predisposes the cells to generate malignant clones [34].

Our study showed a female predominance in both AITD and lymphoma patients, consistent with the common knowledge [18, 20, 21]. However, lymphoma patients in our study presented in the fourth and fifth decades of life, which is quite earlier than reported in previous reports, patients commonly presented in the sixth and seventh decades of life [18, 20, 21]. However, some studies showed similar findings in the same age group. In a retrospective study of patients with MALT lymphoma, the authors reported that the mean age of patients with HT and MALT was 57 (31-80 years) [35]. The detection of this association at this earlier age might be because most patients with HT in our hospital had FNA and thyroidectomy, which diagnose other diseases like lymphomas detected earlier. It was reported that the time interval between the diagnosis of HT and the subsequent development of PTL is in the order of 9-10 years [36], highlighting the importance of early detection and routine investigations.

PTL should always be examined in the differential diagnosis of rapidly growing goiter or thyroid nodules, notwithstanding its rarity. Due to the lack of specificity of any of these for lymphoproliferative diseases, a definitive diagnosis of PTL cannot be made only on the basis of history, physical examination, thyroid function tests, or imaging. For confirmation, tissue is required [37]. FNA has poor reported accuracy for the diagnosis of PTL, despite its importance. According to Hwang and his team, FNA correctly detected PTL in only 60% of individuals who were later shown to have the malignancy [38]. This is due to the histopathology similarities between PTL and HT. The lack of obvious nuclear atypia in PTL makes the cytological distinction between HT lymphoid infiltration and PTL challenging [38–40]. Therefore, some investigators recommended the combination of FNA or ultrasound-guided core needle biopsy in addition to the standard serologic chemistries, serum lactate dehydrogenase, *β*2 microglobulin levels, and core imaging modalities such as fluorodeoxyglucose-PET (FDG-PET) and CT [41–43].

The main modality of treatment in our study was chemotherapy with or without radiation therapy, which is considered the choice for treatment of PTL [31, 44]. In addition to CHOP (cyclophosphamide, doxorubicin, vincristine, prednisolone), the USA-FDA approved the use of rituximab to treat DLBCL. Rituximab is a monoclonal B-cell antibody that specifically binds to the CD20 antigen on pre-B and mature B cells. It was authorized for use with CHOP or other anthracycline-based chemotherapy regimens in patients with DLBCL [45]. When used with CHOP or CVP (cyclophosphamide, vincristine, and prednisone), rituximab improves the outcomes in patients with follicular lymphoma [46]. However, in our study, the follicular lymphoma patient had a slow progressing lymphoma and, therefore, managed with active surveillance. All patients had a complete remission after treatment without any relapses.

The onset of PTL from HT, which is rare, usually follows a chronic transformation over many years; however, some cases have been reported in literature to occur within 18 months after the diagnosis of HT [47]. The occurrence of double malignancies, papillary thyroid carcinoma (PTC), and PTL in HT patients is ever rarer, and in literature, only few cases have been reported in the world so far—from China [48–50], Italy [51], Korea [52], and Peru [53].

This study has some limitations, including the single-center setting and the small sample size, which may hinder the generalizability of our findings. In addition, the cross-sectional design of this study does not denote a causality relationship between the PTL and AITD. Despite these limitations, this is the first study in the Middle East to investigate this association between the PTL and AITD, to the best of our knowledge.

In conclusion, the prevalence of PTL in patients with AITD, specifically HT, was 7%. Most of the patients developed NHL, with DLBCL being the most common subtype. The onset of lymphoma was at a younger age than in literature. All patients with PTL had an HT in their background. Routine investigation in suspected cases with clinical, serological, pathological, and imaging modalities is required to confirm the diagnosis of PTL. It remains unclear whether the diagnosis of AITD increases the risk of developing lymphoma or not. Further national studies are warranted to explore the relation between the two diseases to provide further insight into this association's comprehension.

## Figures and Tables

**Table 1:** Patients' demographics.

	*n*	%
Gender		
Male	9	12.5
Female	63	87.5
Age; mean, SD (43, 14.75)		
Age of diagnosis (years)		
<20	2	2.8
20-29	11	15.3
30-39	19	26.4
40-49	17	23.6
50-59	15	20.8
60-69	3	4.2
70-79	4	5.6
90-99	1	1.4
AITD type		
Graves' disease	14	19.4
Hashimoto thyroiditis	58	80.6
AITD treatment		
Surgery	42	58.3
Surgery and drug	4	5.6
Drug only	16	22.2
Drug and radioiodine treatment	5	6.9
No treatment	5	6.9
Lymphoma type		
Did not develop lymphoma	67	93.1
Developed non-Hodgkin lymphoma	4	5.6
Developed Hodgkin lymphoma	1	1.4
RF: family history		
No	60	83.3
Yes	12	16.7
Total	72	100

GD: Graves' disease; HT: Hashimoto thyroiditis; RAI: radioactive iodine; PTU: propylthiouracil.

**Table 2:** Primary thyroid lymphoma.

Case number	Stage and grade	Specific treatment	Patient status	Remission and relapse
1	Stage I A Hodgkin lymphoma	ABVD × 2, IFRT	Alive	Remission
2	Stage I AE DLBCL grade 3	R-CHOP × 3, IFRT	Alive	Remission
3	Stage II A follicular lymphoma grade 1	Surgery, watchful waiting	Alive	No change in status
4	Stage I AE bulky DLBCL	R-CHOP × 2, IFRT	Alive	Remission
5	Stage II E follicular lymphoma grade 3B	R-CHOP and G-CSF	Alive	Remission

DLBCL: diffuse large B-cell lymphoma; ABVD: Adriamycin, Bleomycin, Vinblastine, Dacarbazine; R-CHOP: rituximab, cyclophosphamide, doxorubicin, vincristine, and prednisone; IFRT: involved-field radiation therapy; XRT: radiotherapy; G-CSF: granulocyte colony-stimulating factor.

**Table 3:** Association of gender with diverse independent variables.

	Male		Female		*p* value
	*n*	%	*n*	%	
AITD type					
Graves	3	21.4%	11	78.6%	0.238
Hashimoto	6	10.3%	52	89.7%	
Total	9	12.5%	63	87.5%	
AITD treatment					0.102
Nothing	0	0.0%	5	100.0%	
Surgery	3	7.1%	39	92.9%	
Surgery and drug	0	0.0%	4	100.0%	
Drug only	5	31.3%	11	68.8%	
Drug and radioiodine treatment	1	20.0%	4	80.0%	
Total	9	12.5%	63	87.5%	
Lymphoma type					
None	8	11.9%	59	88.1%	0.498
Non-Hodgkin	1	25.0%	3	75.0%	
Hodgkin	0	0.0%	1	100.0%	
Total	9	12.5%	63	87.5%	
Lymphoma treatment					
Radiation	8	12.1%	58	87.9%	0.430
Chemotherapy	0	0.0%	2	100.0%	
Surgery	0	0.0%	1	100.0%	
Radiation+chemotherapy	0	0.0%	1	100.0%	
Chemotherapy+surgery	1	100.0%	0	0.0%	
Watchful waiting	0	0.0%	1	100.0%	
Total	9	12.5%	63	87.5%	
Family history (RF)					
No	8	13.3%	52	86.7%	0.534
Yes	1	8.3%	11	91.7%	
Total	9	12.5%	63	87.5%	

Fisher's exact test.

**Table 4:** Association of age at diagnosis with diverse independent variables.

Independent variable	*N*	Mean	SD	*t*	95% CI		*p* value
					Lower	Upper	
Gender							
Male	9	44.33	13.07	0.24	-9.30	11.81	0.813
Female	63	43.08	15.06				
AITD type							
Graves	14	35.14	12.25	-2.36	-18.54	-1.56	0.021
Hashimoto	58	45.19	14.72				
Developed lymphoma							
No	67	42.73	15.06	-1.06	-20.89	6.36	0.291
Yes	5	50.00	7.62				
Family history (RF)							
No	60	43.48	14.70	0.32	-7.88	10.84	0.753
Yes	12	42.00	15.60				
Age 45-50 (RF)							
No	62	41.02	13.97	-3.41	-25.33	-6.64	0.001
Yes	10	57.00	12.20				

Independent *t*-test.

## Data Availability

The data of the manuscript would be available upon request from the corresponding author.
